# MicroRNA-149 suppresses osteogenic differentiation of mesenchymal stem cells via inhibition of AKT1-dependent Twist1 phosphorylation

**DOI:** 10.1038/s41420-021-00618-6

**Published:** 2022-01-10

**Authors:** Jingzhang Fan, Shiming Li, Dawei Wang

**Affiliations:** Department of Orthopedics, The First Affiliated Hospital of Harbin Medical University, Harbin, P.R. China

**Keywords:** Cell biology, Diseases

## Abstract

Osteogenic differentiation is a vital process for growth, repair, and remodeling of bones. Accumulating evidence have suggested that microRNAs (miRNAs or miRs) play a crucial role in osteogenic differentiation of mesenchymal stem cells (MSCs). Hence, the current study set out to elucidate the role of miR-149 in osteogenic differentiation of MSCs and the underlying mechanism. First, rat models of bone differentiation were established using the Masquelet-induced membrane technique, and MSCs were isolated. The expression of miR-149 and AKT1 in the rats and cells was detected with RT-qPCR and western blot analysis. The relationships among miR-149, AKT1, and Twist1 were further predicted by online bioinformatics prediction and verified using dual luciferase reporter gene assay. Alteration of miR-149, AKT1, or Twist1 was performed to further explore their effect on osteogenic differentiation of MSCs. miR-149 was poorly expressed in the process of osteogenic differentiation of MSCs, while AKT1 was highly expressed. miR-149 negatively regulated the expression of AKT1, which in turn diminished the protein levels of Twist1 and promoted the phosphorylation levels of Twist1. Lastly, miR-149 acted as an inhibitor of osteogenic differentiation of MSCs, which could be reversed by AKT1. To sum up, miR-149 silencing promoted osteogenic differentiation of MSCs by enhancing Twist1 degradation through AKT1 upregulation, representing a new method for bone repair treatment.

## Introduction

Mesenchymal stem cells (MSCs) are poly-functional stem cells that normally appear on perivascular regions of various organs [1]. Moreover, MSCs serve as crucial players in cell-based therapies such as metabolic bone diseases, bone regeneration, and bone reconstruction due to their inherent ability to self-renew and their potential of osteogenic differentiation into tissues of mesenchymal origin [2, 3]. Osteogenic differentiation is an intricate biological process that is mediated by both internal cellular signals and external micro-environmental inducements [4]. However, osteogenic differentiation of MSCs is limited for its expensive cost and poor efficiency, which highlights the need for bioactive materials to improve the efficacy of MSC-based therapy regimens [5].

MicroRNAs (miRNAs or miRs) are a group of small regulatory RNA molecules that are implicated in a wide array of processes, including the induction of osteoblast differentiation [6, 7]. For instance, knockout of miR-128 was previously indicated to promote osteogenic differentiation [8]. Meanwhile, miR-125b inhibition has also been proposed as an enhancer of bone defect repair through negative-regulation of osteogenic marker genes in osteogenic differentiation [9]. Moreover, a recent study identified that miR-149-3p possessed the ability to regulate the switch between adipogenic and osteogenic differentiation of bone marrow-derived mesenchymal stem cells (BMSCs), and served as a prospective candidate gene for BMSC-based bone tissue engineering in treating osteoporosis [10]. Furthermore, miR-149 can target the protein kinase B (AKT1) and repress its expression [11]. AKT1, also known as protein kinase B, serves as a central mediator of diverse pathways that regulate cell survival by cell communication [12]. What is interesting is that AKT1 was recently highlighted to function as a specific signaling intermediate in osteoblasts and exhibit a key role in osteoclast differentiation [13]. Meantime, a prior study demonstrated that AKT1 was mediated by miR-19a and thus implicated in the regulation of osteosarcoma metastasis [14]. Besides, AKT1 is capable of enhancing the phosphorylation of twist family bHLH transcription factor 1 (Twist1), which is required for β-TrCP-mediated Twist1 ubiquitination and degradation [15]. Twist1, an essential helix-loop-helix transcription factor, is known to regulate numerous genes related to epithelial-to-mesenchymal transition [16]. In addition, previous research illustrated that the methylation of DNA in the promoter region of Twist1 was a crucial mechanism in osteoblastic differentiation of MSCs [17]. The aforementioned findings and data suggest that miR-149 may be involved in the regulation of osteoblastic differentiation of MSCs by mediating the AKT1/Twist1 axis. Hence, the current study was conducted to verify the interaction between miR-149 and the AKT1/Twist1 axis in the osteoblastic differentiation of MSCs, hoping to provide a theoretical basis for the induction of osteogenic differentiation of MSCs and a novel target for bone repair.

## Results

### Identification of bone differentiation initiated by the Masquelet-induced membrane technique and MSCs in rats

**Fig. 1:**
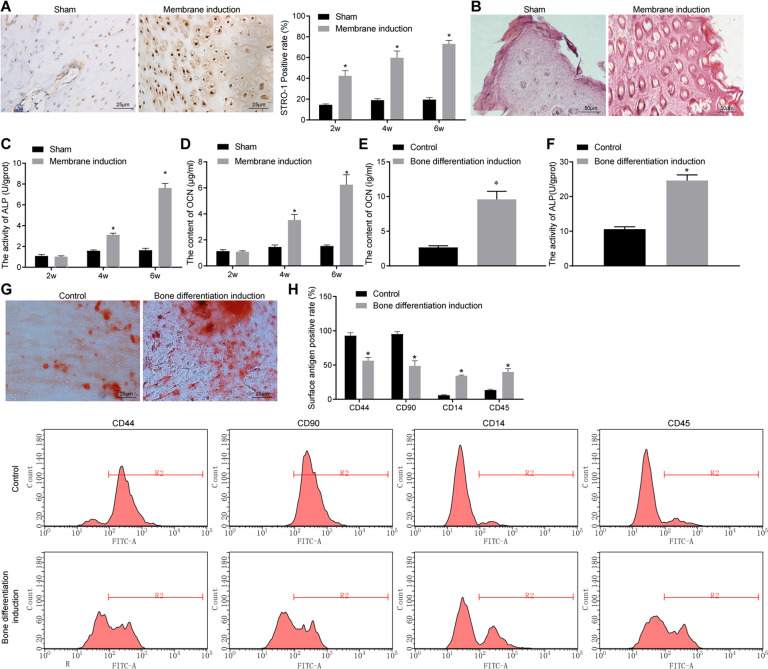
Identification of bone differentiation initiated by the Masquelet-induced membrane technique in rats and MSC isolation. **A** The positive expression rate of STRO-1 in periosteal tissues determined by IHC staining (scale bar = 25 μm). **B** Calcium deposition in periosteal tissues determined by calcified nodule staining (scale bar = 50 μm). **C** ALP activity in periosteal tissues. **D** OCN content in periosteal tissues. **E** OCN content in MSCs. **F** ALP activity in MSCs. **G** Representative images of calcified nodule staining (scale bar = 25 μm). **H** The expression of cell surface differentiation markers CD44, CD90, CD14, and CD45 measured by flow cytometry. *p < 0.05 compared with sham-operated rats or control cells. Data are measurement data and the expressed as mean ± standard deviation, and the unpaired *t* test was used for comparison between two groups. NC negative control, IHC immunohistochemistry, STRO-1 stromal cell antigen 1, ALP alkaline phosphatase, OCN osteocalcin, ANOVA one-way analysis of variance.

In order to establish an effective in vivo research model for experimentation, a rat osteogenic differentiation model was established using the Masquelet-induced membrane technique followed by relevant verification. The periosteum in the wound was subjected to immunohistochemistry (IHC) staining to detect the positive expression rate of stromal cell antigen 1 (STRO-1). It was found that compared with the sham-operated rats, higher expression levels of STRO-1 was found in the periosteum of rats receiving the Masquelet-induced membrane technique, and its expression increased in a time-dependent manner (Fig. 1A). The results of calcified nodule staining revealed that compared with sham-operated rats, calcium deposition was promoted in the periosteum of rats receiving the Masquelet-induced membrane technique (Fig. 1B). Meanwhile, alkaline phosphatase (ALP) activity and osteocalcin (OCN) contents were increased in the periosteum of rats receiving the Masquelet-induced membrane technique compared with sham-operated rats and exhibited significant increases over time (Fig. 1C, D). These findings indicated the successful establishment of bone differentiation rat model using the Masquelet-induced membrane technique.

The isolated MSCs were subjected to control treatment or bone differentiation induction. It was found that ALP activity and OCN content were much higher in cells receiving bone differentiation induction as compared to control cells (Fig. 1E, F). The results of calcified nodule staining illustrated that, compared with control cells, calcium deposition was more pronounced in cells receiving bone differentiation induction (Fig. 1G). In addition, cell surface markers were determined by flow cytometry, and the results demonstrated that CD44 and CD90 were positive, but CD14 and CD45 were negative in cells receiving control treatment. However, CD44 and CD90 were partially positive, whereas CD14 and CD45 were partially negative in cells receiving bone differentiation induction (Fig. 1H). Together, these findings verified the successful isolation of MSCs.

### miR-149 inhibited osteogenic differentiation of MSCs

**Fig. 2:**
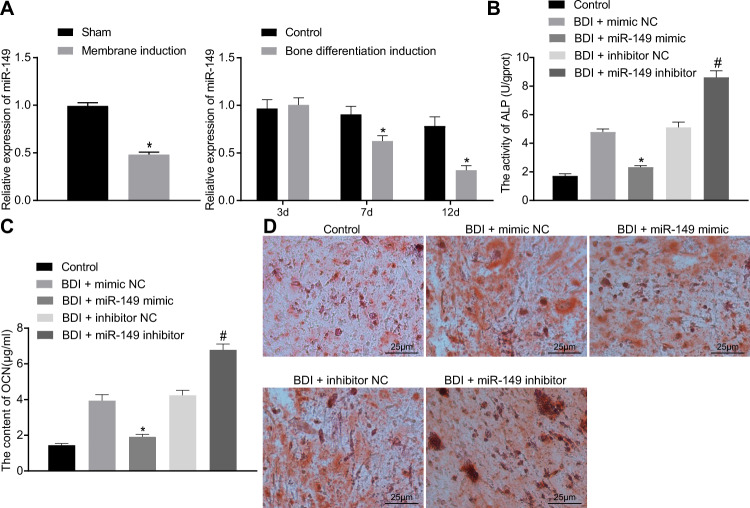
Osteogenic differentiation of MSCs was repressed by miR-149. **A** The expression of miR-149 determined by RT-qPCR, *p < 0.05 compared with sham-operated rats or control cells. **B** ALP activity in MSCs. **C** OCN content in MSCs. **D** Calcium deposition in MSCs determined by calcified nodules staining (scale bar = 25 μm). *p < 0.05 compared with cells after osteogenic differentiation induction treated with mimic-NC, ^#^*p* < 0.05 compared with cells after osteogenic differentiation induction treated with inhibitor-NC. Data were expressed as mean ± standard deviation. Unpaired *t* test was used for data comparison between two groups, and one-way ANOVA was used for data comparison among multiple groups, followed by Tukey’s post hoc test. The experiment was repeated three times. BDI bone differentiation induction, MSC mesenchymal stem cell, ALP alkaline phosphatase, OCN osteocalcin, ANOVA one-way analysis of variance.

To explore whether miR-149 can play a role in osteogenic differentiation, the expression of miR-149 in periosteum of rats after osteogenic differentiation and osteogenic differentiation was also induced in cultured MSCs. Subsequently, the expression of miR-149 was detected by reverse transcription-quantitative polymerase chain reaction (RT-qPCR) on day 3, day 7, and day 12, and the results demonstrated lower expression of miR-149 in rat and cell models of osteogenic differentiation compared to their separate controls (Fig. 2A). Then, to further explore the effect of miR-149 on MSC osteogenic differentiation, we performed bone differentiation induction and intervened the miR-149 expression (Supplementary Fig. 1A). Furthermore, ALP activity and OCN content were found to be significantly reduced in cells transfected with miR-149-mimic, while they were elevated in cells transfected with miR-149-inhibitor (Fig. 2B, C). The results of calcified nodule staining illustrated that calcium deposition was decreased in cells transfected with miR-149-mimic, while those transfected with miR-149-inhibitor presented with significantly increased calcium deposition (Fig. 2D). To sum up, these findings indicated that osteogenic differentiation of MSCs was inhibited by miR-149.

### AKT1 was a target gene of miR-149

**Fig. 3:**
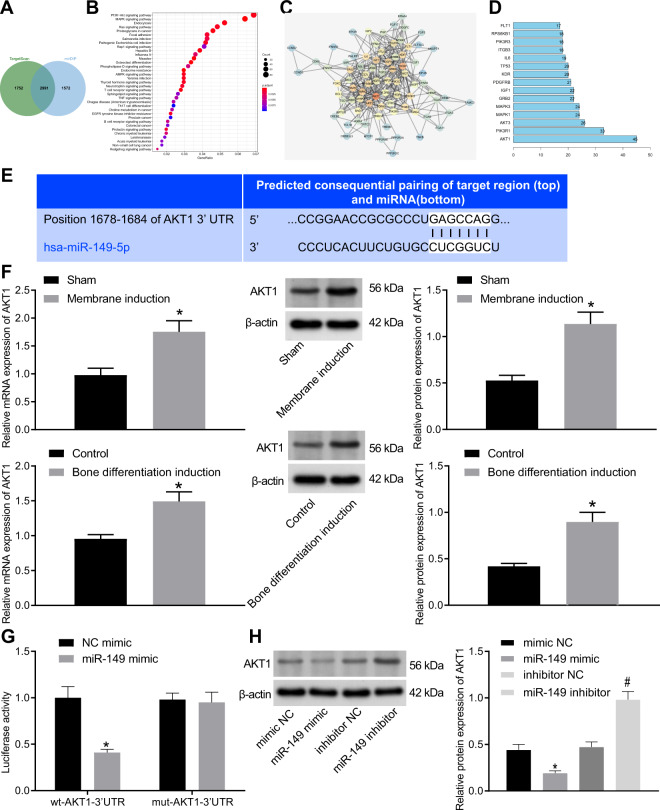
miR-149 targeted AKT1 and repressed the expression of AKT1. **A** The predicted downstream genes of miR-149, the two circles in the figure represented the prediction results of the TargetScan database and the prediction result of the mirDIP database, and the middle part represented the intersection of the two sets of data. **B** The KEGG pathway enrichment analysis of predicted downstream of miR-149, the abscissa in the figure represented the GeneRatio, the ordinate represents the KEGG entry, the size of the circle represents the number of genes enriched in this entry, and the color represented the enrichment *p* value. **C** The interaction analysis of candidate genes that were enriched in the PI3K-AKT signaling pathway, each circle in the figure represented a gene, and the lines between the circles indicated that genes had an interaction relationship. The more interacting genes a gene had, the greater the degree value, the higher the core degree of a gene in the network diagram, the darker the color of the circle. **D** The degree value statistics of the top 15 genes with the highest core degree, the abscissa represented the degree value, and the ordinate represents the gene name. **E** The prediction of the binding between AKT1 and miR-149. **F** The expression of AKT1 in the periosteal tissues of rat receiving the Masquelet-induced membrane technique and cells after osteogenic differentiation, *p < 0.05 compared with sham-operated rats or control cells. **G** The binding of miR-149 to AKT1 detected by dual luciferase reporter gene assay, *p < 0.05 compared with cells transfected with NC-mimic. **H** The protein expression of AKT1 after different treatment determined by western blot analysis, *p < 0.05 compared with cells transfected with mimic-NC, ^#^*p* < 0.05 compared with cells transfected with inhibitor-NC. Data were expressed as expressed as mean ± standard deviation. Unpaired *t* test was used for data comparison between two groups, and one-way ANOVA was used for data comparison among multiple groups, followed by Tukey’s post hoc test. The experiment was repeated three times. ALP alkaline phosphatase, OCN osteocalcin, ANOVA one-way analysis of variance.

In order to explore the downstream regulatory mechanism of miR-149, the downstream genes of miR-149 were predicted using the TargetScan and mirDIP databases, and the prediction results were intersected with 2991 candidate target genes obtained (Fig. 3A). Subsequent Kyoto Encyclopedia of Genes and Genomes (KEGG) pathway enrichment analysis of the obtained candidate genes indicated that they were primarily enriched in the phosphatidylinositol 3-kinase (PI3K)-AKT signaling pathway (Fig. 3B). The candidate genes enriched in the PI3K-AKT signaling pathway were then extracted to conduct gene interaction analysis, and a gene interaction network diagram was plotted (Fig. 3C), with the gene interaction network diagram constructed. It was found that the AKT1 was at the core of the network (Fig. 3D). Online prediction analysis indicated the presence of a specific binding region between the 3’untranslated region (3’UTR) of AKT1 mRNA and miR-149 sequence, and that AKT1 was a target gene of miR-149 (Fig. 3E).

To further explore the role of AKT1 in osteogenic differentiation, the expression of AKT1 in periosteal tissues of rats was detected after osteogenic differentiation. By inducing MSC osteogenic differentiation and measuring the levels of AKT1 in cells on day 7, it was found that compared with sham-operated rats or control cells, AKT1 levels were increased in the periosteal tissues of rats or cells after osteogenic differentiation (Fig. 3F). The results of dual luciferase reporter gene assay showed that compared with cells transfected with mimic-NC, the luciferase activity of the AKT1 3’UTR wild type (WT) was significantly inhibited in cells transfected with mimic-miR-149, while that of AKT1 3’UTR mutant (Mut) exhibited no significant differences (Fig. 3G). In addition, western blot analysis results illustrated that the protein expression levels of AKT1 were notably decreased in response to transfection with miR-149-mimic, while exhibited a marked increase following transfection with miR-149-inhibitor (Fig. 3H). The aforementioned findings demonstrated that AKT1 was a target gene of miR-149 and could be repressed by miR-149.

### miR-149 inhibited osteogenic differentiation of MSCs by regulating AKT1 negatively

**Fig. 4:**
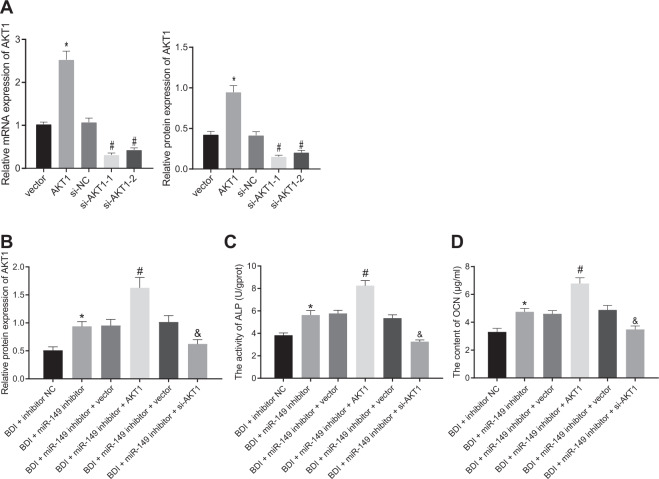
miR-149 suppressed MSC osteogenic differentiation by regulating AKT1. **A** The transfection efficiency of AKT1, *p < 0.05 compared with cells transfected with vector, ^#^*p* < 0.05 compared with cells transfected with si-vector, *n* = 3. **B** AKT levels determined by western blot analysis. **C** ALP activity in MSCs. **D** OCN content in MSCs. *p < 0.05 compared with cells transfected with inhibitor-NC, ^#^*p* < 0.05 compared with cells transfected with miR-149-inhibitor + vector, ^&^*p* < 0.05 compared with cells transfected with miR-149-inhibitor + si-vector, *n* = 3. Data were expressed as mean ± standard deviation. One-way ANOVA was used for data comparison among multiple groups, followed by Tukey’s post hoc test. The experiment was repeated three times. MSC mesenchymal stem cell, ALP alkaline phosphatase, OCN osteocalcin, ANOVA one-way analysis of variance.

To further verify the role of miR-149 regulating AKT1 in osteogenic differentiation, the efficiency of over-expression or downregulation of AKT1 was verified using RT-qPCR and western blot analysis, with small interfering RNA (si)-AKT1 selected for further experiment (Fig. 4A). In addition, the increased expressions of AKT1 after transfection of miR-149-inhibitor were found to exhibit further elevation in MSCs after transfection of over-expression (oe)-AKT1, whereas the increased AKT1 protein levels caused by miR-149 inhibitor were reversed following transfection with si-AKT1 (Fig. 4B). Examination results of ALP activity and OCN content in cells revealed that over-expression of AKT1 brought about an increase in ALP activity and OCN content, while si-AKT1 exhibited the opposite trends (Fig. 4C, D). In short, these findings indicated that osteogenic differentiation of MSCs was repressed by miR-149 by negatively regulating AKT1.

### AKT1 promoted osteogenic differentiation by accelerating Twist1 degradation

**Fig. 5:**
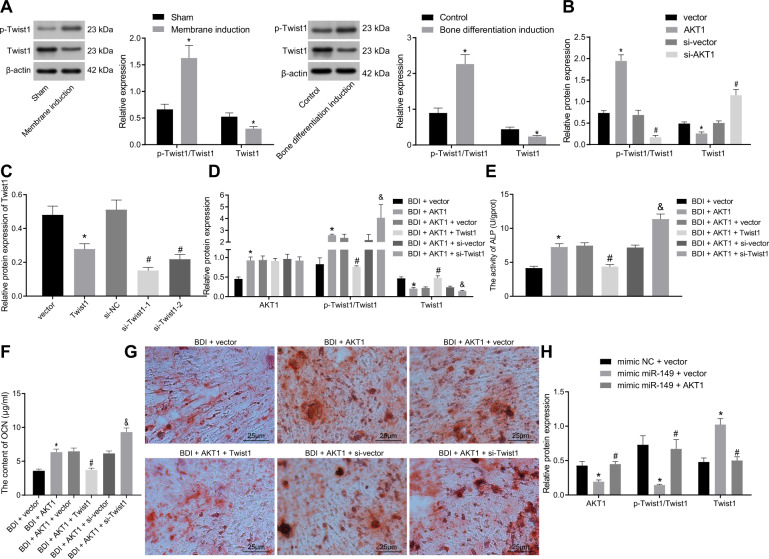
AKT1 facilitated osteogenic differentiation of MSCs by promoting Twist1 degradation. **A** The phosphorylation level of Twist1 and Twist1 protein level determined by western blot analysis, *p < 0.05 compared with sham-operated rats or control cells. **B** The phosphorylation level of Twist1 and Twist1 protein level determined by western blot analysis, *p < 0.05 compared with cells transfected with vector, ^#^*p* < 0.05 compared with cells transfected with si-vector. **C** The expression of Twist1 measured by western blot analysis. **D** The expression of AKT1 and Twist1 determined by western blot analysis. **E** ALP activity in cells. **F** OCN content in cells. **G** Calcium deposition determined by calcified nodule staining (scale bar = 25 μm). *p < 0.05 compared with cells transfected with vector, ^#^*p* < 0.05 compared with cells transfected with AKT1 + vector, ^&^*p* < 0.05 compared with cells transfected with AKT1 + si-vector, *n* = 3. **H** The expression of AKT1 and Twist1 determined by western blot analysis. *p < 0.05 compared with cells transfected with mimic-NC + vector, ^#^*p* < 0.05 compared with cells transfected with mimic-miR-149 + vector. Data were expressed as mean ± standard deviation. Unpaired *t* test was used for data comparison between two groups and one-way ANOVA was used for data comparison among multiple groups, followed by Tukey’s post hoc test. MSC mesenchymal stem cell, ALP alkaline phosphatase, OCN osteocalcin, ANOVA one-way analysis of variance.

To investigate whether the upregulation of AKT1 induced by miR-149 knockout influenced the degradation of Twist1 and thus promoting osteogenic differentiation, the expression of Twist1 was detected in the rat and cell models of osteogenic differentiation. It was found that Twist1 protein levels were decreased, while p-Twist1/Twist1 ratio exhibited a significant increase in the cells or rat models of osteogenic differentiation compared with their separate controls (Fig. 5A). Meanwhile, Twist1 protein levels were decreased in cells following transfection with oe-AKT1, whereas p-Twist1/Twist1 ratios were elevated. However, opposite trends were observed in the cells transfected with si-AKT1 (Fig. 5B).

Subsequently, Twist1-1 was selected for follow-up experiments after the confirmation of the Twist1 over-expression or downregulation efficiency by western blot analysis (Fig. 5C). The results of western blot analysis illustrated that Twist1 over-expression effectively reversed the promotive effect of AKT1 on the p-Twist1/Twist1 ratio and the inhibiting effect on Twist1 protein levels. Meanwhile, combined treatment with si-Twist1 and AKT1 brought about higher p-Twist1/Twist1 ratio and lower Twist1 protein levels than treatment with AKT1 alone (Fig. 5D). Examination of ALP activity and OCN content in cells revealed that Twist1 over-expression reduced the ALP activity and OCN content that were negated following Twist1 silencing (Fig. 5E, F). The results of calcified nodule staining illustrated that over-expression of Twist1 caused a remarkable decrease in calcium deposition, while knockout of Twist1 brought about a marked increase in calcium deposition (Fig. 5G). Furthermore, we found that upregulation of miR-149 induced an increase in the p-Twist1/Twist1 ratio and a decrease in Twist1 protein levels, while simultaneous upregulation of miR-149 and AKT1 elevated the p-Twist1/Twist1 ratio and decreased those of Twist1 protein (Fig. 5H). The above findings supported that miR-149 regulated the degradation of Twist1 through AKT1 to regulate osteogenic differentiation.

### miR-149 inhibited osteogenic differentiation by reducing the degradation of Twist1 via AKT1

**Fig. 6:**
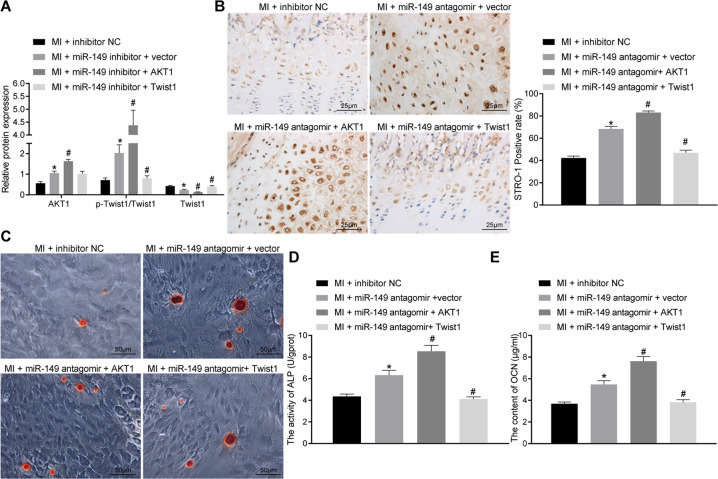
miR-149 repressed osteogenic differentiation by reducing Twist1 degradation through AKT1. **A** The expression of AKT1 and Twist1 in periosteal tissues determined by western blot analysis. **B** IHC staining analysis of STRO-1 protein in periosteal tissues (scale bar = 25 μm) and the quantitative analysis. **C** Periosteum calcified nodule staining (scale bar = 50 μm). **D** ALP activity in periosteal tissues. **E** OCN content in periosteal tissues. *p < 0.05 compared with rats receiving the Masquelet-induced membrane technique treated with inhibitor-NC, ^#^*p* < 0.05 compared with rats receiving the Masquelet-induced membrane technique treated with miR-149-inhibitor + vector. Data were expressed as mean ± standard deviation. Comparison among multiple groups was conducted by one-way ANOVA, followed by Tukey’s post hoc test. MI the Masquelet-induced membrane technique, NC negative control, IHC immunohistochemistry, STRO-1 stromal cell antigen 1, ALP alkaline phosphatase, OCN osteocalcin, ANOVA one-way analysis of variance.

Lastly, we performed in vivo verification of miR-149/AKT1/Twist1 involvement in osteogenic differentiation with the help of rat models. After establishing the rat models of osteogenic differentiation using the Masquelet-induced membrane technique, the expressions of miR-149, AKT1, and Twist1 were altered. Subsequent results of western blot analysis demonstrated that the expression levels of AKT1 and p-Twist1/Twist1 ratio were enhanced, while Twist1 protein levels were decreased in cells following transfection of miR-149-inhibitor, whereas AKT1 reversed protein changes induced by transfection with miR-149-inhibitor (Fig. 6A and Supplementary Fig. 1B). The results of IHC staining analysis illustrated that the positive rate of STRO-1 expression was promoted in the periosteum of rats treated with miR-149 inhibitor, and was further increased by AKT1, but decreased by the treatment of Twist1 (Fig. 6B). Calcified nodule staining results indicated that severe calcium deposition in the presence of miR-149 inhibitor and AKT1 could augment the calcium deposition, whereas Twist1 relieved the calcium deposition (Fig. 6C). In addition, ALP activity and OCN content were found to be increased in cells transfected with miR-149 inhibitor, and were further upregulated by AKT1, but downregulated by Twist1 (Fig. 6D, E). Overall, these findings indicated that miR-149 suppressed osteogenic differentiation by reducing Twist1 degradation through AKT1.

## Discussion

MSCs represent a heterogeneous group of non-hematopoietic progenitor cells, and possess the ability to differentiate into various types of cells such as mesodermal lineages including chondrocytes, adipocytes, osteocytes, myocytes, and endodermal lineages including endothelial cells, keratinocytes [18, 19]. More interestingly, several researchers have indicated the critical role of osteogenic differentiation of MSCs in bone tissue engineering [20]. Similarly, miRNAs are also known to exert crucial functions in controlling osteogenic differentiation of MSCs [21, 22]. In lieu of these facts, the current study performed a series of experiments to explore the effects of miR-149 on osteogenic differentiation of MSCs, and subsequently uncovered that downregulation of miR-149 can enhance the osteogenic differentiation of MSCs by upregulating AKT1 to degrade Twist1.

First, our findings illustrated that miR-149 was downregulated in both rat and cell models of osteogenic differentiation. Subsequently, miR-149 was over-expressed or silenced in cells after osteogenic differentiation induction, which revealed that ALP activity, OCN content, and calcium deposition were all reduced as a result of miR-149 upregulation, while opposing trends were observed after miR-149 silencing, suggesting that miR-149 negatively regulated the osteogenic differentiation of MSCs. Changes in ALP are important due to its role as a crucial biomarker in early differentiation of osteoblasts [23]. Meanwhile, OCN, specifically produced by osteoblasts, is regarded as a key indicator of osteoblast differentiation [24]. On the other hand, miR-206 is known to be poorly expressed in human BMSCs, whereas over-expression of miR-206 can augment ALP activity and mRNA level of OCN, highlighting miR-206 over-expression as a repressor of osteogenic differentiation in BMSCs [25]. Meanwhile, another study highlighted miR-451a knockout as an enhancer of osteogenic differentiation of MSCs, which further promotes bone formation [26]. Furthermore, miR-149 exhibits poor expressions in rats with osteogenic differentiation, whereas ALP activity, OCN content, and calcium deposit were elevated as a result of miR-149 downregulation, which reiterates the promoting role of miR-149 silencing on osteogenic differentiation [27]. Altogether, the aforementioned findings and data indicate that miR-149 can negatively regulate osteogenic differentiation of MSCs.

Additional experimentation in our study revealed that downregulation of miR-149 can augment the expression of AKT1, which further promoted the degradation of Twist1, and ultimately accelerated the osteogenic differentiation of MSCs. Inherently, AKT1 is regarded as a proto-oncogene that is overactive in various cancers, while AKT1 activation requires phosphorylation at Thr308 and further catalytic activity requires phosphorylation at Ser473 [28]. In addition, previous studies have further documented that AKT1 activation exerts a promotive role on the osteogenic activity of Dlx3 by phosphorylating Dlx3, thereby inducing osteogenic differentiation [29]. Moreover, AKT1 can be regulated by miR-149 in glioma, and thus represents a promising target for glioma treatment [30]. In addition, AKT1 was recently indicated to degrade phosphorylation-dependent Twist1, and subsequently inhibited epithelial-to-mesenchymal transition in breast cancer [15]. Twist1 is a primary transcription factor, whose expression can be post-translationally mediated by phosphorylation or ubiquitination activities [31]. Moreover, previous studies have also suggested that decreased mRNA levels of Twist1 contribute to the promotion of osteogenic differentiation [32]. Overall, these findings make it plausible to suggest that Twist1 degradation caused by miR-149 silencing-induced AKT1 upregulation plays a strengthening role in the osteogenic differentiation of MSCs.

To conclude, findings obtained in our study indicate that silencing of miR-149 can facilitate the osteogenic differentiation of MSCs through AKT1-dependent Twist1 degradation. The identification of the miR-149/AKT1/Twist1 axis may facilitate a better understanding of the underlying mechanisms of osteogenic differentiation of MSCs, as well as the development of a potential targets for bone repair in the future. Nevertheless, further investigation into the mechanism is warranted with a diverse study population, so as to support a promising clinical application in treatment for bone injury patients.

## Materials and methods

### Ethics statement

The current study was performed with the approval of the Ethics Committee of The First Affiliated Hospital of Harbin Medical University. All animal experiments were performed in strict accordance with the relevant provisions of the Guiding Opinions on Treating Experimental Animals of the Ministry of Science and Technology of the People’s Republic of China, and conformed to the recommendations in the Guide for the Care and Use of Laboratory Animals published by the US National Institutes of Health. Extensive efforts were made to minimize the number and suffering of the experimental animals.

### Study subject

A total of 14 specific pathogen-free Sprague Dawley (SD) rats (aged 8 weeks old, weighing 300–320 g, purchased from Beijing Vital River Laboratory Animal Technology Co., Ltd., Beijing, China) underwent MI bone differentiation or sham operation, respectively, with 7 rats used for each treatment. The rats were housed at 24–26 °C with relative humidity of 50–70%, with 14/10 h light and dark cycle and allowed free access to food and water.

### Rat model of bone differentiation constructed by the Masquelet-induced membrane technique

Rats were fasted for 24 h but allowed free access to water, and intraperitoneally anesthetized with 3% pentobarbital sodium. The rats were then placed at right decubitus position and their right legs were shaved, and 75% ethanol was used to disinfect the operative area. Next, a longitudinal incision was made in the skin of the lateral femur to incise the skin and fascia, the lateral femoral muscle and biceps femoris muscle were separated bluntly, and the lateral side of the femur was exposed from the greater trochanter of the femur to the femoral condyle. A 6-well stainless steel plate was placed and localized to the center of the lateral surface of femur. After the steel plate was completely fixed, the aforementioned steps were repeated to completely cut off the distal and proximal femurs. The bone defect area in the modeled rats was subsequently filled with polymethyl methacrylate bone cement, while the sham-operated rats were not treated, and the fascia of the two group rats was enwound and sutured, with the operation area rinsed with saline. The muscle and deep fascia were then sutured with Johnson 4-0 Vicryl absorbable sutures, and the skin was sutured with 1-0 silk sutures. Five days after operation, the rats were intramuscularly injected with 80,000 U penicillin (penicillin sodium, North China Pharmaceutical Co., Ltd., Shijiazhuang, Hebei, China, 1.6 million U/bottle, State Food and Drug Administration Approval Number: H13020655) to prevent infection. After subsequent food and water fasting for 12 h, the rats moved with full weight-bearing. The rats were then kept in a single cage for 4 days until the skin incision healed. The fascia was collected and fixed with formaldehyde at the 2nd-, 4th-, and 6th-week time interval after the operation, and the tissues were fixed with decalcification solution for 3 weeks. Later, the decalcification solution was washed, and routinely dehydrated, cleared, and embedded in wax to make paraffin sections for further experimentation.

### IHC staining

Paraffin sections of the tissue samples were dewaxed, dehydrated with alcohol gradient, and then treated with 3% methanol H_2_O_2_ for 20 min and 0.1 M phosphate-buffered saline (PBS) for 3 min, followed by antigen retrieval. Next, the sections were blocked with normal goat serum blocking solution (C-0005, Shanghai Haoranbio Co., Ltd., Shanghai, China) at room temperature for 20 min, and subsequently incubated with primary antibody mouse primary antibody STRO-1 (14-6688-82, dilution ratio of 1:500, Invitrogen, Carlsbad, CA, USA) at 4 °C overnight and then rinsed thrice with 0.1 M PBS (5 min/time). Afterwards, the sections were dilution ratio of incubated with goat anti-mouse secondary antibody IgG (ab205718, 1:20,000, Abcam, Cambridge, UK) at 37 °C for 20 min, rinsed thrice with PBS (5 min/time), and then supplemented with horseradish peroxidase (HRP)-labeled streptavidin protein solution (0343-10000U, Imunbio Co., Ltd., Beijing, China) at 37 °C for 20 min. The sections were later developed with 3,3’-diaminobenzidine tetrahydrochloride (ST033, Whiga Technology Co., Ltd., Guangzhou, Guangdong, China), counterstained with hematoxylin (PT001, Shanghai Bogoo Biological Technology Co., Ltd., Shanghai, China) for 1 min, and then blued in 1% ammonia water. Finally, the sections were dehydrated with certain concentrations of gradient alcohol, cleared with xylene, and sealed with neutral resin before observation and photographing under a microscope.

### RT-qPCR

Total RNA content was extracted from the tissues samples using the TRIzol reagent (15596026, Invitrogen), and then reverse-transcribed into cDNA by a reverse transcription kit (RR047A, Takara, Tokyo, Japan), with a reaction system of 20 μL under reaction conditions of 37 °C for 15 min and 85 °C for 5 s. SYBR Premix EX Taq kits (RR420A, Takara) were employed for sample loading, and the samples were subjected to qPCR reaction in a real-time fluorescence quantitative PCR instrument (ABI7500, ABI, Foster City, CA, USA) with following reaction system: SYBR Mix 9 μL, positive primer 0.5 μL, negative primer 0.5 μL, cDNA 2 μL, RNase Free dH_2_O 8 μL. The reaction conditions were as follows: 95 °C for 10 min, 95 °C for 15 s, and 60 °C for 1 min, for 40 consecutive cycles. Three replicate wells were set for each sample. The primers were synthesized by Shanghai Sangon Biotech company (Shanghai, China), and primer sequences are presented in Supplementary Table 1. Glyceraldehyde-3-phosphate dehydrogenase was uses as the internal reference, and the relative expression of the product was calculated using the 2^-ΔΔCt^ method.

### Western blot analysis

Total protein content was extracted from the tissues or cells using radioimmunoprecipitation assay lysis buffer containing phenylmethylsulphonyl fluoride (P0013C, Beyotime, Shanghai, China), incubated on ice for 30 min, and centrifuged at 8000 g for 10 min at 4 °C, with the supernatant collected. Next, a bicinchoninic acid kit was applied to detect the total protein concentration. After that, the proteins were separated by sodium dodecyl sulfate–polyacrylamide gel electrophoresis and then transferred onto polyvinylidene fluoride membranes using the wet-transfer method. The membrane was subsequently blocked with 5% skimmed milk powder at room temperature for 1 h, incubated with the diluted rabbit primary antibody against AKT1 (56 kDa, dilution ratio of 1:1000, ab81283, Abcam), phosphorylated (p)-Twist1 (23 kDa, dilution ratio of 1:500, ab187008, Abcam), Twist1 (23 kDa, dilution ratio of 1:1000, ab50581, Abcam), and β-actin (43 kDa, ab8227, dilution ratio of 1:2500, Abcam, serving as an internal reference) at 4 °C overnight. Following three rinses with Tris-buffered saline Tween-20 (TBST) (10 min each time), the membrane was incubated with HRP-labeled secondary antibody goat anti-rabbit IgG H & L (HRP) (44 kDa, ab97051, dilution ratio of 1:2000, Abcam) for 1 h. After rinsing with TBST, the membrane was visualized with an enhanced chemiluminescence reagent (Cat. No. BB-3501, Amersham, Little Chalfont, UK). The Bio-Rad image analysis system (Bio-Rad, Hercules, CA, USA) was employed for taking pictures and Quantity One v4.6.2 software was employed for analysis. The ratio of the gray value of the target band to that of β-actin band was representative of the relative protein expression.

### Extraction of rat MSCs

The femurs of both lower limbs of SD rats were isolated under sterile conditions, and the ends of the bilateral stem marrow were excised. Next, the bone marrow cavity was flushed with sterile PBS repeatedly, and the bone marrow was blown out with a sterile syringe to fully disperse the bone marrow cells into a single-cell suspension. The suspension was then filtered through a stainless steel filter to remove blood and bone residue. Afterwards, the cells were collected in a Dulbecco’s modified Eagle’s medium (DMEM)/F-12 medium containing 10% fetal bovine serum (FBS), and then resuspended. The cells were seeded in a culture flask at a density of 1 × 10^6^ cells/mL and cultured in a CO_2_ incubator, with the culture medium changed every 3 days. Upon reaching about 80% confluence, the cells were sub-cultured to obtain MSCs.

### Induction of MSC osteogenic differentiation and cell culture

After transfection, the MSCs were cultured in DMEM/F-12 medium containing 10% FBS in a 5% CO_2_ incubator at 37 °C. The well-grown MSCs of the third generation were seeded in a 6-well plate at a density of 1 × 10^5^ cells/well, and differentiation was induced when the cell confluency reached 70–80%. Next, α-minimum essential medium was added to the cells with 10% FBS, 10 mmol/L β-glycerol phosphate, 10 nmol/L dexamethasone, and 50 μg/mL vitamin C phosphate for osteogenic induction.

With reference to the standard for identification of animal origin MSCs proposed by the Mesenchymal and Tissue Stem Cell Committee of the International Society for Cell Therapy, the cultured BMSCs were identified for cell surface antigens, followed by differentiation. Cells in the control group and the bone differentiation induction group were prepared into a cell suspension with PBS at a density of 1 × 10^6^ cells/L. Next, the cells were incubated in dark conditions with the following fluorescein isothiocyanate (FITC)-labeled rabbit anti-mouse monoclonal antibodies: CD44 (ab25064, Abcam), CD90 (ab25672, Abcam), CD14 (sc-515785 FITC, Santa Cruz Biotechnology, Santa Cruz, CA, USA), CD45 (ab210225, Abcam), 20 μL each. The remaining tube was added with PBS, incubated at room temperature (25 °C) in dark conditions for 30 min, centrifuged, rinsed with PBS, and then regarded as the blank group. Take CD45 expression detection as an example, a forward scatter (FSC)-side scatter (SSC) dot plot was established, with the FSC, SSC voltage, and FSC threshold adjusted to enable the living cell population in the visible range (P1 population) and eliminate cell debris, bubbles, and laser noise interference. A CD45 FITC-SSC scatter plot was established, and the cell population in the P1 gate was displayed. The fluorescence voltage was adjusted to calculate the CD45 positive rate. The positive rates of CD14, CD44, and CD90 were detected as above and a histogram was subsequently plotted.

### Determination of ALP activity and OCN protein level

Cells were lysed with Triton X-100 and centrifuged at 12,000 r/min for 15 min to extract the protein content. In accordance with the instructions of the ALP kit (CD-106854, Wuhan Chundu bio. Com, Wuhan, Hubei, China), the cells were added with 50 μL of ALP substrate reaction solution and color reagent in sequence, and the optical density (OD) value at a wavelength of 405 nm (A405 value) was measured and recorded using a plate reader (CLARIOstar, Biogene Technology Co., Ltd., Hong Kong, China). ALP enzyme activity was determined in cells of the control group, bone differentiation induction group, 5-Aza-CdR group, SDF-1 neutralizing antibody group, and AMD3100 group. This operation was also applicable for tissues.

The cells were collected, lysed, and centrifuged at 3500 r/min for 10 min. Next, 100 μL of supernatant was taken. In accordance with the instructions of rat OCN enzyme-linked immunosorbent assay kit (MM-0622R1, Jingmei Biotechnology Co., Ltd., Jiangsu, China), the OD value at a wavelength of 450 nm was measured using a plate reader (Biogene Technology Co., Ltd.). The OCN protein levels were calculated and recorded.

### Calcified nodule staining

Cell slides were made and after 15 days of culture, the medium was removed, successfully prepared slides were taken and rinsed thrice with pre-chilled PBS, and fixed with 16 g/L paraformaldehyde for 2 h. Following another three rinses with pre-chilled PBS, the cells were added with 50 g/L silver nitrate solution and incubated for 30 min at room temperature in dark conditions. After a wash with double-distilled water, the cells were placed under ultraviolet light for 30 min. Later, the formation of cell mineralized nodules was observed under a microscope and photographed.

### Cell transfection

Exponentially growing cells were seeded in a 6-well plate, and subjected to transfection upon reaching 80% confluence. Briefly, 5 μL Lipofectamine 2000 was added to 250 μL serum-free medium in a tube, mixed and allowed to stand at room temperature for 5 min. Simultaneously, 10 pmol/mL miRNA or mimics was added to another tube. Next, the above two tubes were mixed and left to stand at room temperature for 45 min. Following two washes with Hank’s solution, the cells were added with 1.5 mL of serum-free medium without antibiotics. The above mixture was added to a 6-well plate and shaken gently. Following culture at 37 °C for 4–6 h, the medium was renewed with complete medium and the cells were cultured further for subsequent experiments.

### Bioinformatics analysis and dual luciferase reporter gene assay

The downstream genes of miR-149 were predicted using the TargetScan (http://www.targetscan.org/vert_71/) and mirDIP databases (http://ophid.utoronto.ca/mirDIP/index.jsp#r), followed by the intersection of the obtained prediction results. The “clusterprofile” package of R language was applied to perform KEGG pathway enrichment analysis on the predicted genes. The STRING database (https://string-db.org/) was employed to perform interaction analysis on the predicted genes, and the cytoscapev3.7.1 software was used to construct a gene interaction network diagram. The degree of core genes in the network diagram was counted.

Dual luciferase reporter gene assay was performed to verify the targeting relationship between miR-149 and AKT1. The AKT1 luciferase reporter gene vector and Mut containing miR-149 binding sites were constructed, respectively, namely PmirGLO-AKT1-WT and PmirGLO-AKT1-Mut (designed by Shanghai GenePharma Co., Ltd., Shanghai, China). Next, the luciferase reporter plasmids were co-transfected with miR-149 mimic and mimic-NC plasmids into HEK-293T cells. The cells were collected after 48 h of transfection. In accordance with the instructions of the dual luciferase reporter gene assay kit (D0010, Beijing Solarbio Technology Co., Ltd., China, Beijing, China), the luciferase activity was determined using a Promega’s Glomax 20/20 Luminometer (E5311, Zhongmei Biotechnology Co., Ltd., Shaanxi, China).

### Statistical analysis

Statistical analyses were performed using the SPSS 21.0 statistical software (IBM Corp, Armonk, NY, USA). Measurement data were expressed as mean ± standard deviation. Data comparisons between two groups were conducted by unpaired *t* test, while data comparisons among multiple groups were performed using one-way analysis of variance with Tukey’s post hoc test. A value of *p* < 0.05 was considered statistically significant.

## Supplementary information

Supplementary Figure Legend

Supplementary Figure 1

Supplementary Table 1

## Data Availability

The datasets used and/or analyzed during the current study are available from the corresponding author on reasonable request.
