# Supplementary material for: MicroRNA-149 suppresses osteogenic differentiation of mesenchymal stem cells via inhibition of AKT1-dependent Twist1 phosphorylation
Source: Cell Death Discov. 2022 Jan 10;8:2. doi: 10.1038/s41420-021-00618-6 (PMC8748629; doi:10.1038/s41420-021-00618-6)
Supplement: Supplementary file 1 — Supplementary Figure Legend [file 41420_2021_618_MOESM1_ESM.docx]

**Supplementary Figure Legend**

**Supplementary Fig. 1** Expression of miR-149 in periosteal tissues. A, The expression of miR-149 in periosteal tissues determined by RT-qPCR after bone differentiation induction. B, The expression of miR-149 in periosteal tissues determined by RT-qPCR after membrane induction. * *p* < 0.05 compared with control cells or rats receiving the Masquelet induced membrane technique treated with inhibitor-NC. # *p* < 0.05 compared with cells after osteogenic differentiation induction treated with inhibitor-NC.
