# Supplementary figures and images for: MicroRNA-149 suppresses osteogenic differentiation of mesenchymal stem cells via inhibition of AKT1-dependent Twist1 phosphorylation
Source: Cell Death Discov. 2022 Jan 10;8:2. doi: 10.1038/s41420-021-00618-6 (PMC8748629; doi:10.1038/s41420-021-00618-6)

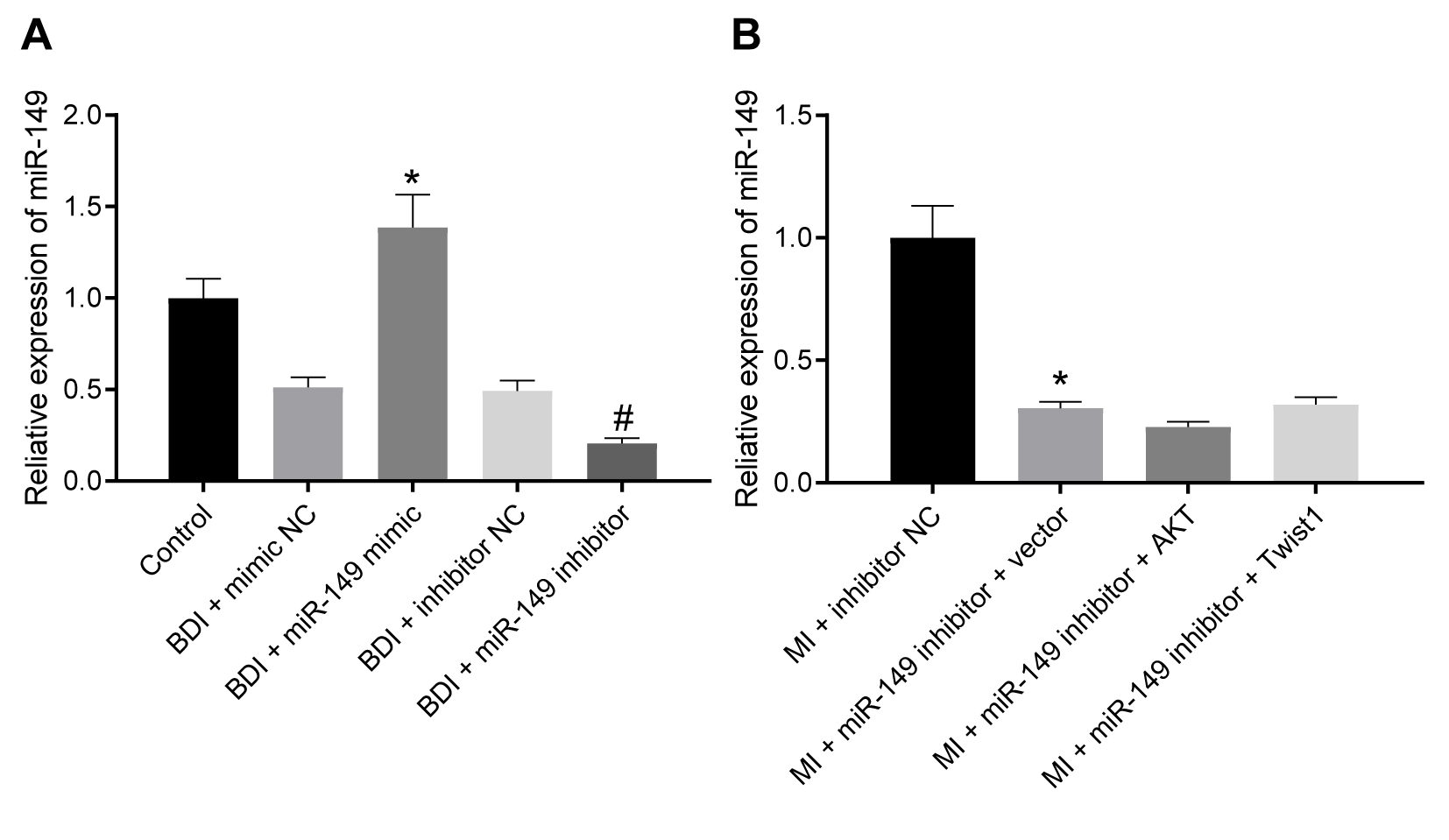

Supplement: Supplementary file 2 — Supplementary Figure 1 [file 41420_2021_618_MOESM2_ESM.tif]
