# Supplementary material for: MicroRNA-149 suppresses osteogenic differentiation of mesenchymal stem cells via inhibition of AKT1-dependent Twist1 phosphorylation
Source: Cell Death Discov. 2022 Jan 10;8:2. doi: 10.1038/s41420-021-00618-6 (PMC8748629; doi:10.1038/s41420-021-00618-6)
Supplement: Supplementary file 3 — Supplementary Table 1 [file 41420_2021_618_MOESM3_ESM.docx]

**Supplementary Table 1** Primer sequences for RT-qPCR

| Target | Primer sequence |
| --- | --- |
| miR-149 (rat) | F: 5’-TCTGGCTCCGTGTCTTCACTCCC-3’ |
|  | R: 5’-AGTGGTTGTTCTGCTCTCTGTGTC-3’ |
| GAPDH (rat) | F: 5’-GAAGGTGAAGGTCGGAGTC-3’ |
|  | R: 5’-GAAGATGGTGATGGGATTTC-3’ |
| U6 (rat) | F: 5’-AAAGCAAATsCATCGGACGACC-3’ |
|  | R: 5’-GTACAACACATTGTTTCCTCGGA-3’ |
| AKT1 (rat) | F: 5’-CTGAGATTGTGTCAGCCCTGGA-3’ |
|  | R: 5’-CACAGCCCGAAGTCTGTGATCTTA-3’ |

Note: miR-149, microRNA-149; GAPDH, glyceraldehyde-3-phosphate dehydrogenase; RT-qPCR, reverse transcription-quantitative polymerase chain reaction; F, forward; R, reverse.
